# On the Connexion between Morbid Physical and Religious Phenomena

**Published:** 1856-07-01

**Authors:** J. F. Denham


### Art. V.—ON THE CONNEXION BETWEEN MORBID PHYSICAL AND RELIGIOUS PHENOMENA.

BY THE REV. J. F. DENHAM, M.A., F.R.S., ETC.

[No. VI. of a Series.]

In several former papers of this series, references have been made to the subject of Introspection; as being both an effect and reacting cause of bodily disease; and as being combined with morbid religious and even moral phenomena, and as the indirect cause of such phenomena. This subject will also be still more largely referred to in future papers; and especially in the next of them, Introspection will be considered as one of the chief proximate causes of moral evil. I beg, therefore, to offer a complete explanation of my views of it on the present occasion, distributed under the following particulars:—I. The definition of introspection. II. Proofs that it is not the normal condition of the mind, but the effect of bodily disease, and that the indulgence of it augments bodily disease. III. A description of the morbid religious phenomena with which it is frequently attended. IV. A specification of the chief mischiefs resulting from it. V. The suggestion of some means for its prevention and cure.

I. Definition. By introspection is meant, as the etymology of the term imports, the act or habit of looking within; and it is now to be understood as the attention of the mind being turned inward upon itself; or, to the contemplation of its own real or supposed phenomena, and especially of what are commonly called its own feelings. It is briefly, the state of the mind's *positive consciousness of itself.*

II. The following proofs are offered in support of the conclusion that introspection as now defined is (1) not the normal condition of the mind; but that (2) it is a result and reflex cause of bodily disease. The most natural, and therefore the best condition of the mind, and in reference to its *entire* agency, is that of subconsciousness, or of a consciousness which although constantly possessed, and accompanying all the mind's present operations,* is not perceived by the mind itself; does not rise so high as to obtrude itself on the mind's own attention. This normal condition of consciousness, I shall beg leave to call the negative state of the faculty; and, on the contrary, to affix the name of positive consciousness to that state of it in which it becomes so active, or rises so high, as more or less to occupy, and therefore to embarrass, the mind's attention.

And with regard to the intellectual part of our nature, all persons

* Stewart's "Outlines of Moral Phil." Edin. 1829. Sec. 1—9.

are aware that all their best mental operations are conducted during the negative state of the consciousness; when, to use popular language, "we have become lost," or "have forgotten ourselves," in our subject or object:—that is, when our consciousness, though still alive, is not so active as to be perceived by us, and therefore, does not clog the operation of any of our faculties or powers. Thus, we never think or write, or speak so well, as when, along with a sufficient acquaintance with our subject, we have ceased to be aware that we are so employed. We even learn best when we are least conscious that we are learning. Every teacher knows the importance of inducing self-forgetfulness in the pupil, because no progress can be made until this oblivion of self is established. With regard also to our moral powers, we are never so virtuous and truthful as when we speak and act without having a distinct idea that we are virtuous and truthful. The same remarks apply also to our religious and pious capabilities. On the contrary, if we become conscious that we are thinking upon any subject, the process of thought becomes instantly perturbed, or suspended—if that we are speaking, we hesitate—if that we are acting in a just and upright manner, our moral fabric undergoes a tremor—if that we are pious, our piety partakes of formality and effort. These observations may be extended to our most habitual, and even to our automatic actions. Thus, if we become conscious of the action of breathing, nictitation, walking, &c., these become either suspended, or performed in a less regular and efficient manner. Even in regard of our bodily existence in general, or of any of its particular parts, it is well known that the highest state of health is that of a negative consciousness—or when we do not find our attention called to the existence or action of any portion of our frame—but that, on the contrary, whenever we become aware that we have a head, heart, stomach, foot, &c., that is, whenever a positive state of the consciousness in regard of any of our members, &c., begins, it is a certain intimation of some injury, weakness in them, diseased state, or disordered action. It would seem, then, that the most perfect action of all our intellectual, moral, and religious powers, as of our physical, ever goes on in a degree below our consciousness of it; and that, on the contrary, a state of positive consciousness, by whatever means produced, indicates a wrong action of that portion of our nature to which it belongs. In fewer words, excellence of all kinds is unconscious of its own existence—a maxim that may be illustrated in all the various departments of talent, beauty, virtue, piety, &c. In still fewer words, self-consciousness spoils action.

I crave permission to introduce, at this stage of my observations, the fullest and best reference to the subject of introspection that

I have met with, taken from a work* which I may quote without thereby involving an entire acquiescence with all its contents, by the Rev. Chauncy Hare Townshend, A.M., late of Trinity Hall, Cambridge.

"Consciousness is susceptible of various developments, which have never yet been properly distinguished into their several grades. Metaphysicians have done no more than enumerate the simple consciousness of the moment, and remembered consciousness. They have omitted a third action of this faculty—namely, reflective consciousness, or internal observation, which is one of the operations of consciousness; and, not being identical with its parent, should not be involved with it in one common definition. Simply to feel, or simply to pass again through a succession of former feelings with a sense of their relation to one personal identity, is not the same as to be self-regardant and watchful of our sensations as they arise. Under the last circumstances the mind is manifestly in another state and tone of feeling. Its state is then, that of introspective consciousness—or the mind's action when self-regardant. It has *ourselves* for its object. It varies in degrees, from the constant self-scrutiny, both mental and physical, that some persons carry on in society, when they observe their every least word, fearful to utter aught amiss, and their own least gesture, lest they should commit an awkwardness—to the unmixed and simple consciousness of reverie. Would we find man's distinguishing stamp of mental superiority, we must seek it in that abstraction in which the pure intellectuality reigns alone, and almost free from any disturbance of the introspective consciousness, which being of itself an act, annihilates, pro tempore, all other acts. Were we perpetually to exercise the reflex act of the mind—and to pause upon our thoughts with self-observation, our train of ideas would halt, and fall to pieces for want of connexion.

"But this is not all. Any admixture of the introspective consciousness detracts from the perfection of one's acquired and habitual motions, as much as it spoils the freedom and bold expansion of our thoughts. Of this we may soon convince ourselves. Though generally insensible of the act of breathing, we may, by attention, become aware of the process. What follows? An immediate sense of uneasiness, and interruption of that regular motion which seems to go on so well of itself. Again, that winking of the eye, whereby the organ is healthily preserved, becomes a torment if we think about it. Again, too, every musician must have felt that, when he has learnt to play a piece of music by heart, if he *thinks* upon the direction of his fingers, he is apt to play false. Let him trust to the simply memorial consciousness of his physical being, and he does not err. Again, the operations of memory are impeded by the introspective consciousness, as Darwin, in his *Zoonomia,* observes,—'We frequently experience, when we are doubtful about the spelling of a word, that the greater voluntary exertion we use, that is, *the more intensely we think about it*, the further we are from regaining the lost association, which readily

* "Facts in Mesmerism," &c. Second edition, 1844, p. 201, &c.

recurs when we have become careless about it.' Introspective consciousness, then, appears equally to mar our liberty and our memory, both of thought and action; and consequently it should seem that, in proportion as we can be exempted from its interference, we must attain a higher state of intellect, and of corporeal activity. This we may surmise; but proof is not wanting to confirm it. The state of the philosopher who solved the problem of the universe, was avowedly a state of abstraction, and of self-forgetfulness; and it is equally well known that natural sleep-walkers who can never be supposed capable of self-scrutiny, will achieve feats which would be the horror of their waking hours. They will stand, self-balanced, on the ridge of a house, where, under the usual conditions of consciousness, they could not preserve their equilibrium for a single moment. They will cross a roaring torrent on a single plank—but if suddenly awaked to a contemplation of themselves, or their situation—they will lose their footing or perhaps die of alarm. Are these examples too far removed from general experience? We will, then, bring the matter at once home to every man's personal feelings. What is it that accompanies and adds to the awkwardness caused by timidity? An overwatchfulness, a care that mars itself—in fine—the too predominant presence of the introspective consciousness. The shy scarcely ever forget themselves, as it is called—make them do so, and their deportment is at once improved. In proportion as introspectiveness is annulled, the powers of thought and motion are developed."

It seems also deserving of notice that the evils of introspection are recognised in the instinctive language and ideas of the uneducated classes, when they speak of "thinking too deeply about things," "brooding over our thoughts," &c., "letting things take hold of one," "the thoughts preying upon themselves," "laying things to heart," "falling down upon oneself;" and when they advise those suffering from such a state of consciousness to "divert their thoughts to other things," to "look outward rather than inward"—which last advice is often given, from necessity, by that class of religious teachers whose instructions chiefly tend to awaken positive consciousness in their hearers, in order to prevent the formidable consequences sometimes resulting from such instructions from reaching a disastrous extreme. It is also worthy of remark that the frequent comment of the healthy-minded portion of the community on the victims of morbid religious feelings is, that such persons are "always thinking about themselves."

If the foregoing proofs, &c., be deemed sufficient to render probable the position that introspection, or positive consciousness, or that state of the mind in which it is self-regardant, or its attention is directed to itself, to its own existence, perceptions, feelings, &c., is not the normal condition of the mind, because it suspends, or perturbs, or perverts the mind's action—

the conclusion seems to follow that (2) such a condition of the consciousness is the result of disease; and since, further, it is the opinion of the highest medical authorities that the state of our intellectual functions depends chiefly upon the condition of the nervous power,* the inference seems safe that a tendency to introspection originates in some kind or other of bodily disease immediately or ultimately affecting the brain,—and thereby the mind,—whether the disease be constitutional or self-induced, whether chronic or temporary, and whether originating in ideas first addressed to the mind, such as erroneous religious instruction acting upon an infirm or morbid temperament, &c., or in the indulgence of introspection as a morbid gratification, or the cultivation of it as a mistaken religious duty. The proof of the morbid physical origin of introspection might indeed be rested on our experience or observation, from which we learn that neither ourselves nor others are prone to this state of mind, except along with some conscious disease or disorder of the vital organs.

I would here beg to resume a principle frequently propounded in my preceding papers—that the body—the entire physical constitution—may be the origin or source, according to its particular state in regard of health, not merely of corresponding feelings, &c., but also of conceptions, ideas, and trains of thought, and that a morbid physical state or action, and especially of the brain, heart, stomach, liver, bowels, &c., excites, perhaps primarily through morbid sensations, a corresponding set of morbid thoughts, perceptions, reasonings, and imaginations, in all their alternations and variety—or, to use the words of Gaubius, "the mind perceives differently according to the various conditions of the body to which it is joined, and she may be disturbed by the body in her operations, and at some times be hindered from thinking as she would, and at other times be compelled to think as the body commands."†

III. It is now proposed to describe the morbid religious phenomena sometimes attending introspection. The patient's attention is more or less concentrated on himself, and on what is called, in the language of a certain religious school, his "*experience*"—that is, upon the state of his feelings, or, to speak more strictly, on the suggestions arising from his feelings to his mind. Even his countenance, attitude, and manners, but especially a peculiar introverted expression of the eyes, indicate that the process of his auscultation to his own inward feelings, &c., is

* Cullen's "First Lines of the Practice of Physic," book iv., ch. i., paragraphs 1540-1.

† "Philosophical Discourse on the Management and Cure of the Disorders of the Mind," by H. D. Gaubius. Translated by J. Tapprell, M.D.

going on. Very often he endeavours to excite in himself what he considers a desirable state of feelings, which he still mistakes for ideas; and not a few persons succeed for a time in the endeavour, by directing the action of the mind to some part of the frame, chiefly the stomach, and other viscera; and from whose morbid action the coveted feeling may be excited by the proficient almost at will. As might be expected, the ideas he obtains from such feelings, whether of an elated or despondent nature, are irrational, and after passing through various alternations, end in a settled vapidity, occasioned, as it should seem, by the exhaustion of the physical organs, &c. He then experiences constant dissatisfaction with himself and with everything he does, complains that he cannot feel, or "realize," religious truths or objects, finds no "evidences" within himself of his acceptance with God, but is full of unbelief and guilt, that his mind is cold or dark, or that his soul is beset with horrid suggestions. These, and all other morbid religious phenomena, are always attended with similar phenomena in regard of *other* subjects and objects: thus the patient is also *otherwise* excited or despondent, suspicious, distrustful of himself, and incapable of sound mental exertion, and even of correct moral feeling. The usual course of the disease is its mitigation along with returning health, and increase along with the increase of bodily ailment; it becomes chronic when associated with chronic disorder, and along with the decay of the physical powers terminates in fatuity.

IV. The mischiefs resulting from introspection include all the evils that can arise from the disturbance or suspension of all the powers and faculties of our nature; but in particular cases will depend upon the degree to which it is exercised, and the extent and nature of the physical disease, organic or functional, with which it is combined; nor can it be doubted but that the indulgence of it, in consequence of directing the mind's attention to the diseased physical state or action, increases such state or action, so that the ill habit of mind and body co-act and increase each other; and the very texture of that part of the body and the perverted action of the mind upon it may become reciprocally both cause and effect. Besides those evils already adverted to under previous particulars, introspection is the especial parent of indecision, uncertainty, and scepticism, which may proceed to the extent of a total loss of confidence in all sensations, perceptions, and principles. Such an effect in the department of religion is thus described by the judicious Hooker:—

"Men may many times in judgment of themselves be so confounded, that they find not themselves in themselves. For that which dwelleth in their hearts they seek, they make diligent search and inquiry. It abideth, it worketh in them, yet still they ask where; still they lament as for a thing which is past finding: they mourn as Rachel, and refuse to be comforted, as if that were not which indeed is, and as if that which is not were; as if they did not believe when they do, and as if they did despair when they do not; which in some, I grant, is but a melancholy passion, proceeding only from that dejection of mind, the cause whereof is the body, and by bodily means can be taken away. . . . They fasten their suggestions upon the distrustful cogitations of the flesh, whereof finding great abundance in themselves, they gather thereby. But tell this to a man that hath a mind deceived by too hard an opinion of himself, and it doth but augment his grief: he hath his answer ready, 'Will you make me think otherwise than I find, than I *feel in myself?* I have thoroughly considered and *exquisitely* sifted all the corners of my heart, and I see what there is. Never seek to persuade me against my knowledge. 'I do not, I know I do not, believe.' "*

I would suggest, for the consideration of the reader, whether the ancient Pyrrhonism and the absurdities of scepticism in all ages may not have arisen from a like cause? It may also be remarked that introspection may be a conducing cause of moral evil. To me it seems certain that no moral evil can arise without a previous act or habit of this reflex act of the mind, that the origin of vice and crime is the "manet altâ mente repostum," the "flammato secum corde volutans," the "imo pectore," the "intus," so often referred to as such by the Roman poets. The Scriptures also thus describe "the wicked" and "the workers of iniquity:" "They search out, or imagine wickedness; both the inward thought of every one of them, and the heart is deep."† It is "the imagination of the thoughts of man's heart that is only evil continually."‡ It is "when lust hath conceived that it bringeth forth sin."§ Certainly introspection is one cause of idleness, the acknowledged origin of all sins, or rather it is the pernicious employment to which idleness resorts, and out of which it fabricates its mischiefs. Hence, too, most likely arose the Christian maxim to lay the check upon the thoughts, or, to use its own expression, upon "the heart as the origin of all evil things."|| It would also seem to be the excellence of Christianity, that, in regard of its facts, duties, worship, and expectations, it is not a contemplative religion; and, I may be permitted to remark, upon the same quality as attending the practical expression of Christianity embodied in the Liturgy and offices of the Church of England.

The liability to the peculiar kind of hypochondria, as, perhaps, the physician would call it, which I have denominated introspection, may, no doubt, as already intimated, be partly ori-

* Works, vol. ii. pp. 592-593. Oxford, 1845. † Psalm lxiv. 5, 6.
‡ Genesis vi. 5. § James i. 15. || Matthew xv. 19.

ginated or augmented by those books or that kind of preaching which inculcate an unsparing and critical examination, not of "our lives and conversations by the rule of God's commandments," but of the internal phenomena and feelings—under the name of *self-examination;* and I beg leave to offer a refutation of that misuse of the Scriptures upon which such a self-examination is commonly founded. The passages usually urged are the two following: "Let a man examine himself, and so let him eat of that bread and drink of that cup:"* and "Examine yourselves, whether ye be in the faith; prove your own selves."† If, however, these passages, and any other frequently adduced for the same purpose, be interpreted according to their context, scope, and occasion, no direction can be fairly derived from them for that analysis of the mental phenomena, feelings, &c., involved in the process of introspection. A reference to any respectable commentary will show that St. Paul, in the first of these passages, requires the Corinthians only to examine themselves "whether they partook of the Lord's Supper as a common meal, or as the bond of a faction, or to promote some worldly purpose;"‡ and that in the second of these passages, the apostle directs the Corinthians to "judge by the miraculous gifts among them, and which St. Paul had himself imparted to them, whether Christ spoke in, or by him, or not,"—"simply to ask themselves."§ It is worthy of notice, that the apostle declined to "judge himself, but left the judgment of himself to the Lord;"‖ and that the psalmist asks of *God* to "examine him and know his heart; to try him and know his thoughts; and see if there were any wicked way in him, and to lead him in the way everlasting."¶ It is also remarkable that the nearest approach to introspection recorded in the Scriptures terminated in an unfavourable result. It is that of the psalmist, who "communed with his own heart, and made diligent search," or, as in the Prayer-book version, "searched out his own spirits," and who consequently became bewildered and despondent, and lost all faith in the divine mercy, and at length ascribes his state of mind to "his own infirmity," and adopted the better method of considering "the works" and the recorded acts of the Almighty, as the means of his consolation.**

There is, also, extreme danger attending the attempt to raise within ourselves an intense perception of any of the realities of our creed,—to "realize" such things, as it is called,—because, it being impossible to know when such a perception is adequately raised, the mind, habituated to the attempt at raising it, strains

* 1 Cor. xi. 28. † 2 Cor. xiii. 5.

‡ Macknight. § Whitby. ‖ 1 Cor. iv. 3, 4.

¶ Psalm cxxxix. 23, 24. ** Psalm lxxvii.

its own powers to an unlimited extent, and ultimately undermines its own energies.

It was, no doubt, a sense of the general evils resulting from introspection that induced "the self-torturing sophist" Rousseau, who was himself a miserable sufferer from them, to maintain that it is so far opposed to our nature, that "the man that reflects is a monster." It must, however, be allowed, that the most correct, as well as most serviceable, of all our perceptions,—intellectual, moral, and religious,—are those which first and almost immediately arise in the mind; and that, on the contrary, those which are the result of the most laborious thought and intense feeling are generally the most absurd, and even immoral. It is part of the great poet's description of the dangerous man, that "he thinks too much;"* and certainly, the best disposed and conducted persons are those who do not scrutinize their thoughts and feelings too deeply.

V. The prevention and cure of a tendency to introspection, considered as the result of bodily disease, must be sought—primarily, at least—in the care and restoration of the bodily health. Along with medical means appropriate to the case, the utmost care should be taken to prevent the mind's attention from being turned inward upon itself, or upon its own thoughts, &c., by directing it entirely to outward and interesting objects and engagements, and to a diversity of them in a natural but unbroken succession. Idleness ought to be especially avoided, as one chief cause of this malady, and particularly in the case of bodily disorder—of which, however, it is both the effect and cause. The natural liability of the mind to become disordered when disengaged, is evident even in the cessation of its activity preliminary to sleep, and during imperfect sleep itself. Hence the poet who best understood human nature, whether sane or diseased, puts the prayer into the lips of a good man,—

> "Merciful powers,
> Restrain in me the cursed thoughts, that nature
> Gives way to in repose."†

It is, also, sometimes requisite to forbid the study of mental philosophy to such persons as are prone to introspection, and to which study they are often inclined, and who commonly imagine that they are studying the phenomena of the mind, while, in fact, they are only contemplating their own morbid feelings. No persons, indeed, under an inferior state of health, should indulge reflection, which, even in the best state of health, generally injures both mind and body, and too frequently raises a host of irrelevant ideas, and useless, if not pernicious, feelings. When

* "Julius Cæsar," Act i., Scene 11. † "Macbeth," Act ii., Scene 1.

carried beyond due bounds its results may be formidable. I find two cases in my memoranda, both of them of persons of learning and talent,—the one of a man who meditated on death till he acquired the fixed idea that he possibly had already died, and was already in the state of existence after death; and the other, of a man who, by too long and profoundly studying the subject of the Divine existence and attributes, became tormented with the propensity to ask why he himself was not the Divine Being. Certainly it is preferable to be "fools of nature," than

> "So horridly to shake our disposition,
> With thoughts beyond the reaches of our souls."*

It is also advisable to prohibit the perusal of religious biographies, which too frequently excite an inclination, or a sense of duty, especially in hypochondriac temperaments, to cultivate a tone of sentiment and motive never really possessed by any human being. Nor less needful is it to inculcate a virtuous satisfaction with the attainment of ordinary virtues, and the due performance of common duties. The victims of introspection not unfrequently are plagued with an ambition

> "For that goodness, which, growing to a pleurisy,
> Dies in its own too-much."†

It is in consequence of this emulation of ideal excellence, co-operating with disease of the physical constitution, that the anguish of a mere intense consciousness may be mistaken for the action of conscience, contrition, or humility, but whose unfailing result is merely religious indolence or despair. All stimulants and excitements, physical and mental, should be avoided, for they all have a tendency to heighten the self-consciousness; and in this quality consists their attraction and their danger. The author of the "Natural History of Enthusiasm" remarks on the too great possibility of "rushing from the scenes of an excited devotion to the chambers of filthy sin."‡ It should be the constant object of us all to use the means of grace simply as means, and with faith in their efficacy, and to take more care of our conduct than of our feelings, for these will generally take the direction, good or evil, given them by our practical demeanour. It is also a good rule to turn the attention from the object or idea that excites the undue action of the consciousness to that undue action itself; for when the mind is drawn off from the object or subject to attend to its own operation, that operation ceases and escapes our notice; and, what is equally valuable, we also forget the subject or object.§

* "Hamlet," Act i., Scene 4.
† "Hamlet, Act iv., Scene 7.
‡ Isaac Taylor.
§ Reid, Essay i., ch. vi., sec. 4.

